# Noise correlation and its impact on the performance of multi‐material decomposition‐based spectral imaging in photon‐counting CT

**DOI:** 10.1002/acm2.13830

**Published:** 2022-11-17

**Authors:** Xiangyang Tang, Yan Ren, Huiqiao Xie

**Affiliations:** ^1^ Imaging and Medical Physics, Department of Radiology and Imaging Sciences Emory University School of Medicine Atlanta Georgia USA

**Keywords:** CT, material‐specific imaging, multi‐material decomposition, noise correlation; noise, photon‐counting CT, spectral CT, virtual monochromatic imaging

## Abstract

**Purpose:**

It has been known that noise correlation plays an important role in the determination of the performance of spectral imaging based on two‐material decomposition (2‐MD). To further understand the basics of spectral imaging in photon‐counting CT toward optimal design and implementation, we study the noise correlation in multi‐MD (*m*‐MD) and its impact on the performance of spectral imaging.

**Method:**

We derive the equations that characterize the noise and noise correlation in the material‐specific (basis) images in *m*‐MD, followed by a simulation study to verify the derived equations and study the noise correlation's impact on the performance of spectral imaging. Using a specially designed digital phantom, the study of noise correlation runs over the cases of two‐, three‐, and four‐MD (2‐MD, 3‐MD, and 4‐MD). Then, the noise correlation's impact on the performance of spectral imaging in photon‐counting CT is investigated, using a modified Shepp–Logan phantom.

**Results:**

The results in 2‐MD show that, in‐line with what has been reported in the literature, the noise correlation coefficient between the material‐specific images corresponding to the basis materials approaches −1. The results in *m*‐MD (*m* ≥ 3) are more complicated and interesting, as the noise correlation coefficients between a pair of the material‐specific images alternate between ±1, and so do in the case of 4‐MD. The *m*‐MD data show that the noise in virtual monochromatic imaging (a form of spectral imaging) is moderate even though the noises in material‐specific (basis) images vary drastically.

**Conclusions:**

The observation of noise correlation in 3‐MD, 4‐MD, and beyond (i.e., *m*‐MD) is informative to the community. The relationship between noise correlation and the performance of spectral imaging revealed in this work may help clinical medical physicists understand the fundamentals of spectral imaging based on MD and optimize the performance of spectral imaging in photon‐counting CT and other X‐ray imaging modalities.

## 1 INTRODUCTION

As a quantitative imaging method that is well balanced over its contrast, spatial and temporal resolutions, CT has become one of the most widely used modalities for diagnostic imaging in clinics, with more than 80 million procedures carried out annually in the United States alone.^1^ However, the challenges in clinical applications are demanding CT to be more potent in contrast resolution for early detection and characterization of pathologies in soft tissues. Owing to its capability of material‐specific and virtual monochromatic imaging, the spectral imaging implemented in photon‐counting CT offers the opportunity for scientists and researchers in the field to make CT more potent in detection and characterization of vascular and parenchymal lesions, especially in concert with administration of contrast agents, either state‐of‐the‐art iodinated^2^ or other novel contrast agents^3^ in the future.

Contrast agent has been indispensable in conventional CT since the very beginning and will continue to be the case in photon‐counting CT, especially in light of the prospect that novel contrast agents in the form of biodegradable nanoparticles may play more important roles. The spectral imaging in photon‐counting CT with administration of one or multiple contrast agents^4^ demands in‐depth studies of multi‐material decomposition (*m*‐MD). Recently, based on an in‐depth revisiting of the physics underlying photon‐counting CT, we have carried out studies on the condition of basis materials (functions) and spectral channelization (energy binning) for spectral imaging. Using the approach based on singular value decomposition and analysis,^5^, ^6^, ^7^, ^8^ we have shown how the condition of basis materials and spectral channelization can affect the performance of spectral imaging in photon‐counting CT^2^, ^3^ based on multi‐MD (*m*‐MD). In this work, we address another fundamental issue in photon‐counting CT ‐ the correlation of noise and its impact on the performance of spectral imaging.

It has been known from the beginning that the noise in the projection data acquired at two distinct peak tube voltages are of relevance in determining spectral CT's imaging performance.^8^, ^9^ With resort to maximum likelihood estimation, Alvarez and Macovski showed that the Jacobian of the transform from projection space to *A*‐space^10^ determines the noise property that is governed by the Cramér–Rao lower bound.^5^ Alternatively, Kelcz et al. studied the same issue by taking noise as the uncertainty in estimating the basis materials’ mass density.^11^ Using the first‐order Taylor expansion, Kelcz et al. found that the so‐called *R*‐quantity, which is the ratio of mass attenuation coefficients associated with the two interested materials at the two distinct peak voltages, plays a key role that is almost identical to the Jacobian of the transform from projection space to *A*‐space.^12^

Initially, Alvarez and Macovski assumed no correlation in noise between the projection data acquired at the two peak voltages (correlation‐vanishing henceforth), as this is the cases in data acquisition implemented via photon‐counting under ideal spectral response or voltage‐switching (termed as fast kV‐switching nowadays).^8^, ^9^, ^13^ They showed that though the noise correlation in projection data vanishes, there still exists a negative noise correlation in the image corresponding to each basis material^8^, ^9^, ^13^, ^14^ (material‐specific image henceforth). Notably, the assumption of correlation‐vanishing seems invalid in the case of data acquisition via layered (sandwiched) detector. However, through an in‐depth statistical analysis, it turns out that the noise correlation in the data acquired by the first and second layers is in fact negligibly small and thus can be treated as another case of correlation‐vanishing.^15^ Nevertheless, the correlation‐vanishing assumption is actually not valid in the data acquisition by photon‐counting if realistic detector response is considered, as the spectral distortion inevitably induces correlation in the projection data acquired at the low and high peak voltage.^5^, ^6^, ^7^

In spectral imaging based on 2‐MD, an investigation to reveal the effect of noise correlation in the projection data on noise and noise correlation in the material‐specific images has been reported in the literature.^16^ Analytically, it was shown that the correlation between the noise in the two material‐specific images is negative, and, if the correlation in the projection data vanishes, the analytics degenerates to the case that was initially derived by Alvarez and Macovski.^8^, ^9^, ^13^ Notably, the work in Ref. [^16^] was focused on the counting‐integration mode in data acquisition, and only analytic plots were given, though the conclusion is in fact general and applicable to other cases of 2‐MD‐based spectral imaging implemented via different schemes of data acquisition.^16^

With the engagement of multispectral channels and multi‐basis materials, the dimension of Jacobian increases accordingly. This case, in turn, complicates the way at which the noise correlation in projection data acquired in each spectral channel impacts the noise correlation in the material‐specific images. Specifically, the sign of the off‐diagonal elements in the inverse of an *M* × *M* (*M >* 2) matrix may vary, implying that the noise correlation corresponding to each material‐specific image in the *m*‐MD‐based spectral imaging may not be always negative, as has been the case in 2‐MD‐based spectral imaging.^14^ Via analytical derivation, phantom studies, and quantitative analysis, we have investigated the noise correlation in the material‐specific (basis) images via *m*‐MD and reported the preliminary data.^17^ In this work, we extend our effort to further investigate the noise correlation and study its impact on the performance of *m*‐MD‐based spectral imaging in photon‐counting CT, in which we focus on the cases of *m*‐MD (*m* ≥ 3) that are carried out in the projection domain, with the goal to provide the information for further understanding of the basics of *m*‐MD‐based spectral imaging in photon‐counting CT and other X‐ray imaging. Purposely, our work is designed to be a phantom study supported by computer simulation, so that any adverse effects, which may be induced by the imperfection in photon‐counting CT's imaging chain, on the investigation and its conclusion can be ruled out.

## 2 METHOD

### 2.1 Modeling of signal detection and material decomposition in photon‐counting CT

The modeling of signal and its detection in photon‐counting CT have been detailed in our recent publication^5^, ^6^, ^7^ and are only reiterated here briefly for reader's convenience. If the noise in data acquisition is taken into consideration, the detection of signal in photon‐counting CT can be analytically expressed as

(1) $$\begin{array}{ccc} & & I_{k}\left(L\right)=\\ & & \mathrm{Poisson}\left(\int_{E_{\min}}^{E_{\max}}D_{k}\left(E\right)N_{0}\left(E\right)\exp\left(-\int_{L}\mu \left(x;E\right)\mathrm{dl}\right)\mathrm{dE}\right) \end{array},$$

where $N_{0}\left(E\right)$ denotes the spectrum of X‐ray source, and *L* is the path penetrated by the X‐ray beam. Subscript *k* indexes the spectral channel in which the raw data are acquired, whereas $D_{k}\left(E\right)$ is defined in the *k*th spectral channel [$E_{min}^{k}E_{max}^{k}$] that takes detector's efficiency *η*(*E*) and spectral response into account.^5^, ^6^, ^7^ *Poisson*(*⋅*) denotes the numerical operation that generates random numbers to observe the Poisson distribution.^18^In MD, we can write

(2) $$\mu \left(x;E\right)=\sum_{i=1}^{K}a_{i}\left(x\right)\mu_{i}\left(E\right).$$

Then, Equation (1) is converted into

(3) $$\begin{array}{c} I_{k}\left(L\right)=\mathrm{Poisson}\left(\int_{E_{\min}}^{E_{\max}}D_{k}\left(E\right)N_{0}\left(E\right)\exp\left(-\sum_{p=1}^{P}A_{p}\left(L\right)\mu_{p}\left(E\right)\right)\mathrm{dE}\right) \end{array}$$

Given *p* = 1, 2, …, *P*, $A_{p}\left(L\right)=\int_{L}a_{p}\left(x\right)\mathrm{dl}$ denotes the line integral of $a_{p}\left(x\right)$ associated with the mass distribution of material *p*, which can be obtained from $I_{k}\left(L\right)$ (*k* = 1, 2, …, *K*) by solving the set of integral equations specified in Equation (3) through numerical methods, for example, the iterative Newton–Raphson algorithm,^9^, ^19^ in which the integral equations’ initial conditions are obtained by system calibration via polynomial data fitting.^9^, ^19^, ^20^

### 2.2 Data acquisition and image formation in photon‐counting CT

**FIGURE 1:**
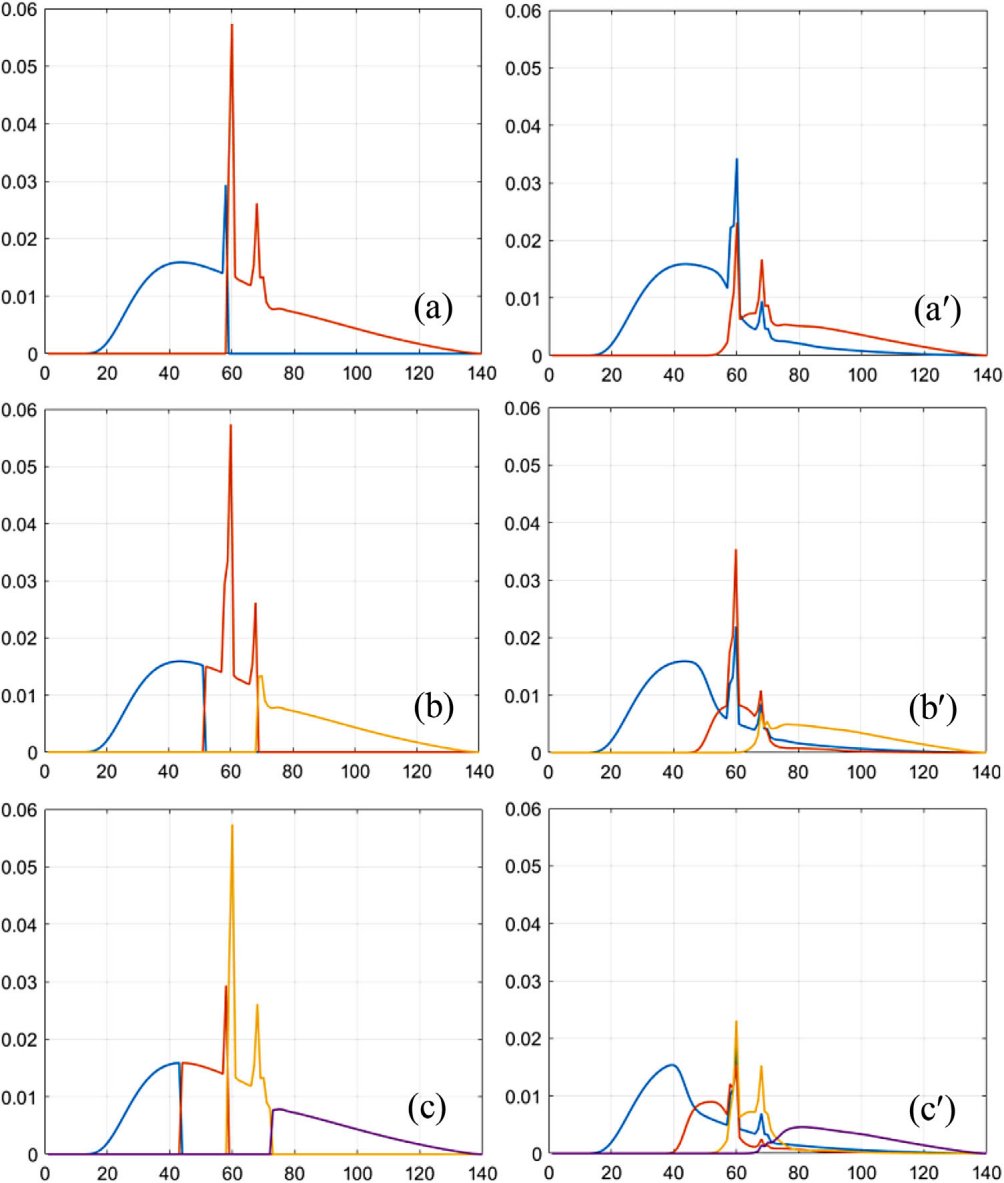
Two (a), three (b), and four (c) spectral channels under ideal detector spectral response and their counterparts (a′–c′) under realistic detector response (keV is the unit of abscissa)

In simulation,^21^ the X‐ray technique is set at 1000 mA*/*140 keV, and the gantry of photon‐counting CT rotates at 1 rot/s. The spectral channels are defined as [1–58, 59–140] keV in 2‐MD (Figure 1a,a′), [1–51, 52–68, 69–140] keV in 3‐MD (Figure 1b,b′), and [1–43, 44–58, 59–72, 73–140] keV in 4‐MD (Figure 1c,c′). The goal in spectral channelization is to ensure that the photon counts in each channel be about the same at the incident surface of the object to be imaged, in spite of other criteria that have been used in practice.^22^ For illustration, the spectral channels under ideal detector response are plotted in Figure 1a–c, whereas their counterparts under realistic detector response in Figure 1a′–c′. The material in the photon‐counting detector assumed in the simulation is cadmium zinc telluride (CZT) or cadmium telluride, which is associated with the Compton scattering, charge‐sharing, and fluorescent‐escaping that may distort the detector's spectral response.^6^, ^7^ The photon‐counting detector is designed as a curved two‐dimensional array at 864 × 16 dimension and 1.024 × 1.092 mm^2^ pitch. The source‐to‐iso and source‐to‐detector distances are 541.0 and 949.0 mm, respectively, consistent to one of the multi‐detector row CT scanners in the clinic. After MD, each basis image $a_{p}\left(x\right)$ is in turn reconstructed from $A_{p}\left(L\right)$ (*p* = 1, 2, …, *P*) via an FBP image reconstruction algorithm.^23^

### 2.3 Noise propagation in *m*‐MD‐based spectral imaging

By treating *I_k_(L)* (*k* = 1, …, *K*) and *A_p_(L)* (*p* = 1, …, *P*) as random variables in the projection space and *A*‐space, respectively, the mapping from the former to the latter can be written as

(4) $$I_{k}\left(L\right)=f_{k}\left(A_{1}\left(L\right),\cdots ,A_{p}\left(L\right)\right)\left(k=1,\cdots ,K\right).$$

Then, we have the covariance matrix of *A* as^16^, ^24^

(5) $$V\left[A\right]=\left(F^{-1}\right)\cdot V\left[I\right]\cdot {\left(F^{-1}\right)}^{T},$$

where each entry of the mapping matrix *F* is defined as $F_{kp}=\partial I_{k}/\partial A_{p}$. In analog to the way in Ref. [16], we define the effective attenuation coefficient, signal‐to‐noise ratio (SNR), correlation coefficient in the projection space, and *A*‐space, respectively, as

(6) $$\mu_{\mathrm{kp}}=-\frac{1}{I_{k}}\frac{\partial I_{k}}{\partial A_{p}},\mathrm{SN}R_{k}=\frac{I_{k}}{\sigma_{I_{k}}},$$

(7) $$\rho_{I_{k1}I_{k2}}=\frac{\sigma_{I_{k1}I_{k2}}}{\sigma_{I_{k1}}\sigma_{I_{k2}}},\rho_{A_{p1}A_{p2}}=\frac{\sigma_{A_{p1}A_{p2}}}{\sigma_{A_{p1}}\sigma_{A_{p2}}}.$$

#### 2.3.1 Noise propagation in 2‐MD‐based spectral imaging

In 2‐MD, we have the Jacobian

(8) $$\Delta_{2\times 2}=\mu_{11}\mu_{22}-\mu_{21}\mu_{12}\Delta =\frac{1}{A_{1}A_{2}}det\left(F\right).$$

According to Equation (5), the covariance matrix *V*[*A*] becomes

(9) $$V_{2\times 2}\left[A\right]=\left[\begin{array}{cc} \sigma_{A_{1}}^{2} & \sigma_{A_{1}A_{2}}\\ \sigma_{A_{2}A_{1}} & \sigma_{A_{2}}^{2} \end{array}\right],$$

where the entries are

(10) $$\begin{array}{c} \sigma_{A_{1}}^{2}=\frac{1}{\Delta_{2\times 2}^{2}}\left(\frac{\mu_{22}^{2}}{\mathrm{SN}R_{1}^{2}}+\frac{\mu_{12}^{2}}{\mathrm{SN}R_{2}^{2}}-2\rho_{I_{12}}\frac{\mu_{22}}{\mathrm{SN}R_{1}}\frac{\mu_{12}}{\mathrm{SN}R_{2}}\right), \end{array}$$

(11) $$\sigma_{A_{2}}^{2}=\frac{1}{\Delta_{2\times 2}^{2}}\left(\frac{\mu_{21}^{2}}{\mathrm{SN}R_{1}^{2}}+\frac{\mu_{11}^{2}}{\mathrm{SN}R_{2}^{2}}-2\rho_{I_{12}}\frac{\mu_{11}}{\mathrm{SN}R_{2}}\frac{\mu_{21}}{\mathrm{SN}R_{1}}\right),$$

(12) $$\begin{array}{ccc} \sigma_{A_{1}A_{2}} & = & \sigma_{A_{2}A_{1}}=-\frac{1}{\Delta_{2\times 2}^{2}}\left(\frac{\mu_{21}\mu_{22}}{\mathrm{SN}R_{1}^{2}}+\frac{\mu_{12}\mu_{11}}{\mathrm{SN}R_{2}^{2}}\right.\\ & & \left.-\;\rho_{I_{12}}\left(\frac{\mu_{11}}{\mathrm{SN}R_{2}}\frac{\mu_{22}}{\mathrm{SN}R_{1}}+\frac{\mu_{12}}{\mathrm{SN}R_{2}}\frac{\mu_{21}}{\mathrm{SN}R_{1}}\right)\right), \end{array}$$

Furthermore, we have the cross‐correlation

(13) $$\rho_{A_{1}A_{2}}=\rho_{A_{2}A_{1}}=\frac{-\left(\frac{\mu_{21}\mu_{22}}{\mathrm{SN}R_{1}^{2}}+\frac{\mu_{12}\mu_{11}}{\mathrm{SN}R_{2}^{2}}-\rho_{I_{12}}\left(\frac{\mu_{11}}{\mathrm{SN}R_{2}}\frac{\mu_{22}}{\mathrm{SN}R_{1}}+\frac{\mu_{12}}{\mathrm{SN}R_{2}}\frac{\mu_{21}}{\mathrm{SN}R_{1}}\right)\right)}{{\left(\frac{\mu_{22}^{2}}{\mathrm{SN}R_{1}^{2}}+\frac{\mu_{12}^{2}}{\mathrm{SN}R_{2}^{2}}-2\rho_{I_{12}}\frac{\mu_{22}}{\mathrm{SN}R_{1}}\frac{\mu_{12}}{\mathrm{SN}R_{2}}\right)}^{1/2}{\left(\frac{\mu_{21}^{2}}{\mathrm{SN}R_{1}^{2}}+\frac{\mu_{11}^{2}}{\mathrm{SN}R_{2}^{2}}-2\rho_{I_{12}}\frac{\mu_{11}}{\mathrm{SN}R_{2}}\frac{\mu_{21}}{\mathrm{SN}R_{1}}\right)}^{1/2}}$$

If there is no correlation between the projection data acquired at the two peak voltages, the following equations degenerate into

(10′) $$\sigma_{A_{1}}^{2}=\frac{1}{\Delta_{2\times 2}^{2}}\left(\frac{\mu_{22}^{2}}{\mathrm{SN}R_{1}^{2}}+\frac{\mu_{12}^{2}}{\mathrm{SN}R_{2}^{2}}\right),$$

(11′) $$\sigma_{A_{2}}^{2}=\frac{1}{\Delta_{2\times 2}^{2}}\left(\frac{\mu_{21}^{2}}{\mathrm{SN}R_{1}^{2}}+\frac{\mu_{11}^{2}}{\mathrm{SN}R_{2}^{2}}\right),$$

(12′) $$\sigma_{A_{1}A_{2}}=-\frac{1}{\Delta_{2\times 2}^{2}}\left(\frac{\mu_{21}\mu_{22}}{\mathrm{SN}R_{1}^{2}}+\frac{\mu_{12}\mu_{11}}{\mathrm{SN}R_{2}^{2}}\right),$$

(13′) $$\rho_{A_{1}A_{2}}=\frac{-\left(\frac{\mu_{21}\mu_{22}}{\mathrm{SN}R_{1}^{2}}+\frac{\mu_{12}\mu_{11}}{\mathrm{SN}R_{2}^{2}}\right)}{{\left(\frac{\mu_{22}^{2}}{\mathrm{SN}R_{1}^{2}}+\frac{\mu_{12}^{2}}{\mathrm{SN}R_{2}^{2}}\right)}^{1/2}{\left(\frac{\mu_{21}^{2}}{\mathrm{SN}R_{1}^{2}}+\frac{\mu_{11}^{2}}{\mathrm{SN}R_{2}^{2}}\right)}^{1/2}},$$

which is exactly the same as that given by Alvarez and Macovski at the very beginning.^8^, ^9^ In general, an interchannel spectral overlapping under realistic detector spectral response induces positive correlation between the data acquired in each channel, that is, $\rho_{I_{12}}>0$, which decreases the magnitude of nominators in Equations (10)–(13). Meanwhile, however, the interchannel overlapping may decrease the magnitude of $\Delta_{2x2}^{2}$ in the denominator to a larger extent. Jointly, these changes may increase the magnitude of noise and noise correlation in the data denoted by *A*_1_ and *A*_2_ that are obtained via MD.

#### 2.3.2 Noise propagation in 3‐MD‐based spectral imaging and beyond

In 3‐MD, we have the Jacobian

(14) Δ3×3=μ11μ22μ33+μ12μ23μ31+μ21μ32μ13−μ31μ22μ13−μ32μ23μ11−μ11μ21μ33Δ=1A1A2A3detF.

Again starting from Equation (5), the covariance matrix *V*[*A*] becomes

(15) $$V_{3\times 3}\left[A\right]=\left[\begin{array}{cc} \begin{array}{c} \sigma_{A_{1}}^{2}\\ \sigma_{A_{2}A_{1}} \end{array} & \;\begin{array}{cc} \sigma_{A_{1}A_{2}} & \;\sigma_{A_{1}A_{3}}\\ \sigma_{A_{2}}^{2} & \;\sigma_{A_{2}A_{3}} \end{array}\\ \sigma_{A_{3}A_{1}} & \;\begin{array}{cc} \sigma_{A_{2}A_{2}} & \;\sigma_{A_{3}}^{2} \end{array} \end{array}\right],$$

with the entries being derived in the Appendix. Similarly, the interchannel spectral overlap induces positive correlation between the data acquired in different channels, that is, $\rho_{I_{12}}>0$, $\rho_{I_{23}}>0$, and $\rho_{I_{13}}>0$, which, in general, decreases the magnitude of nominators in Equations (A1)–(A6). Moreover, the interchannel overlap may decrease the magnitude of $\Delta_{3x3}^{2}$ in the denominator to a larger extent. Jointly, these changes may increase the magnitude of noise and noise correlation in the data denoted by *A*_1_–*A*_3_ after MD.

As shown in the Appendix, the analytic expression of each entry of matrix *V*_3×3_[*A*] becomes exhaustively complicated in 3‐MD, though their derivation can be carried out according to Equation (5). Hence, an analysis of the noise and noise correlation via simulation study makes more sense in terms of feasibility in the case of 4‐MD‐ (and beyond) based spectral imaging in photon‐counting CT.

### 2.4 Analysis of noise correlation and imaging performance via simulation study

The noise correlation and its impact on the performance of spectral imaging are assessed via simulation study, in which the imaging chain of photon‐counting CT is modeled and simulated using a software kit.^5^, ^6^, ^7^, ^21^

#### 2.4.1 Analysis of noise correlation

**FIGURE 2:**
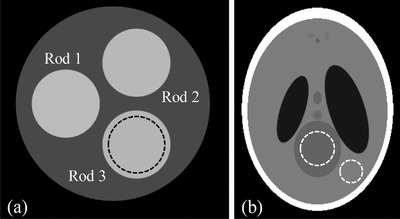
Sectional view of the noise correlation phantom (a) and the head phantom for assessment of imaging performance (b)

A cylinder of water at 20 cm diameter, which consists of three rods at 7 cm diameter, is designed as the phantom to assess the correlation of noise in material‐specific images in photon‐counting CT (henceforth, the noise correlation phantom). The materials that make rods 1–3 corresponding to the cases of 2‐MD, 3‐MD, and 4‐MD are listed in Table 1. Specifically, two materials (soft tissue and cortical bone) are assumed in the 2‐MD case, whereas iodine and gadolinium are added into the cases of 3‐MD and 4‐MD, respectively. The fraction of each material in the rods are chosen for quantitative purpose without any implication toward clinical or preclinical applications, as such the difference in material composition is adequate to ensure the accuracy in quantitative measurement. A sectional view of the phantom is presented in Figure 2a, in which a region of interest (ROI) at 6 cm diameter within rod 3 is defined for illustration of noise and noise correlation measurement.

**TABLE 1:** Material configuration (fraction in weight) of the three rods in the phantom used for evaluation and verification of noise correlation in the material‐specific (basis) images in 2‐material decomposition (MD), 3‐MD, and 4‐MD, respectively

	2‐MD	3‐MD	4‐MD
Rod 1	1/3 soft tissue 2/3 cortical bone	2/7 soft tissue 4/7 iodine (10 mg/ml) 1/7 cortical bone	2/7 soft tissue 2.5/7 iodine (10 mg/ml) 2/7 gadolinium (10 mg/ml) 0.5/7 cortical bone
Rod 2	1/2 soft tissue 1/2 cortical bone	3/7 soft tissue 3/7 iodine (10 mg/ml) 1/7 cortical bone	3/7 soft tissue 2/7 iodine (10 mg/ml) 1.5/7 gadolinium (10 mg/ml) 0.5/7 cortical bone
Rod 3	2/3 soft tissue 1/3 cortical bone	4/7 soft tissue 2/7 iodine (10 mg/ml) 1/7 cortical bone	3/7 soft tissue 2.5/7 iodine (10 mg/ml) 1/7 gadolinium (10 mg/ml) 0.5/7 cortical bone

A number of basis materials have been investigated in our previous studies.^5^, ^6^, ^7^ In the study of analyzing noise correlation, we choose soft tissue and cortical bone as the basis materials for 2‐MD^28^, and soft tissue, 10 mg/ml gadolinium, and cortical bone for 3‐MD. In the case of 4‐MD, the basis materials are chosen as soft tissue, 10 mg/ml iodine, 10 mg/ml gadolinium, and cortical bone.

Once the material‐specific images are generated, ROIs are drawn in each of them as illustrated in Figure 2a. The Pearson correlation coefficient (*R*‐coefficient), as defined in the following, is adopted to quantitatively assess the correlation of noise in those images:

(16) $$R=\frac{\sum_{i=1}^{N}\left(x_{i}-\bar{x}\right)\left(y_{i}-\bar{y}\right)}{\sqrt{\sum_{i=1}^{N}{\left(x_{i}-\bar{x}\right)}^{2}}\sqrt{\sum_{i=1}^{N}{\left(y_{i}-\bar{y}\right)}^{2}}},$$

where $x_{i}$ and $y_{i}$ denote the pixels at identical location in each of the two material specific images. $\bar{x}$ and $\bar{y}$ are the averaged intensities of all the pixels in each of the two ROIs, and *N* is the total number of pixels in each ROI.

#### 2.4.2 Assessment of spectral imaging performance

A humanoid head modified from the Shepp–Logan phantom (henceforth the head phantom) is used to study the noise correlation's impact on the performance of virtual monochromatic imaging (a form of spectral imaging) in photon‐counting CT. Presented in Figure 2b is a sectional view of the head phantom, wherein two ROIs are defined as the areas of signal (big dashed circle) and background (small dashed circle) for measurement of contrast, noise, and contrast‐to‐noise ratio (CNR):

(17) $$\mathrm{CNR}=\frac{\left(m_{\mathrm{sig}}-m_{\mathrm{bkg}}\right)}{\frac{1}{2}\left(\sigma_{\mathrm{sig}}+\sigma_{\mathrm{bkg}}\right)},$$

where $m_{sig}$ and $\sigma_{\mathrm{sig}}$ are the mean and standard deviation gauged in the signal ROI, and $m_{bkg}$ and $\sigma_{\mathrm{bkg}}$ are the counterparts in the background ROI. The mass attenuation coefficients of the materials in the head phantom are determined according to authoritative publications, for example, Refs. [25, 26] and the EPDL library.^27^ The readers who are interested in the configuration details of the phantom are referred to Refs. [6, 7]. The study of noise correlation's impact on the performance of spectral imaging is focused on the 3‐MD case, in which four sets of basis materials are selected: (i) BM‐1: adipose, PMMA, and Teflon; (ii) BM‐2: adipose, PMMA, and 20 mg/ml iodine; (iii) BM‐3: soft tissue, 20 mg/ml iodine, and cortical bone; (iv) BM‐4: adipose, 20 mg/ml iodine, and Teflon.

## 3 RESULTS

### 3.1 Noise and noise correlation in 2‐MD material‐specific images

**FIGURE 3:**
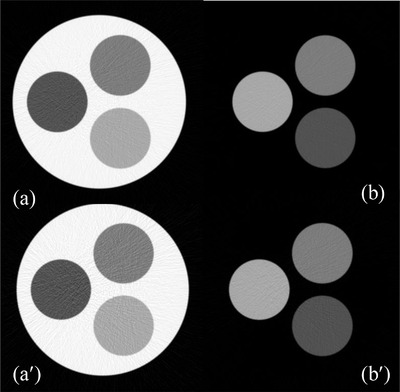
Material‐specific (tissue [a,a′] and bone [b,b′]) images acquired via two‐material decomposition (2‐MD), whereas the detector response is ideal (top) or realistic (bottom).

**FIGURE 4:**
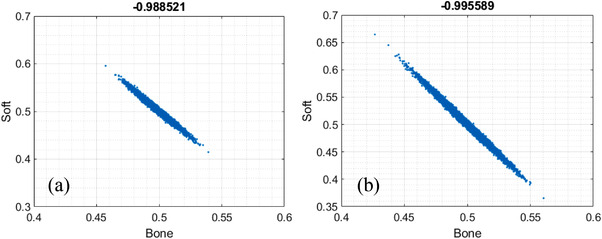
Correlation of noise between material‐specific images acquired via two‐material decomposition (2‐MD) under ideal (a) and realistic (b) detector spectral response

The material‐specific images corresponding to basis materials soft tissue and cortical bone obtained via 2‐MD under ideal and realistic detector spectral response are presented in the top and bottom rows of Figure 3, whereas the noise correlation corresponding to these two material‐specific images are displayed in the scatterplot in Figure 4a,b, respectively. The noise in the material‐specific images under 2‐MD is in negative correlation, which is consistent to what has been reported in the literature^13^, ^14^, ^16^ and serves as a validation of the MD implemented in our study. Moreover, it is observed that, in addition to increasing the magnitude of noise in each material‐specific image markedly (Figure 3), the interchannel overlapping in detector's spectral response increases the magnitude of noise correlation coefficient modestly (Figure 4).

### 3.2 Noise and noise correlation in 3‐MD material‐specific images

**FIGURE 5:**
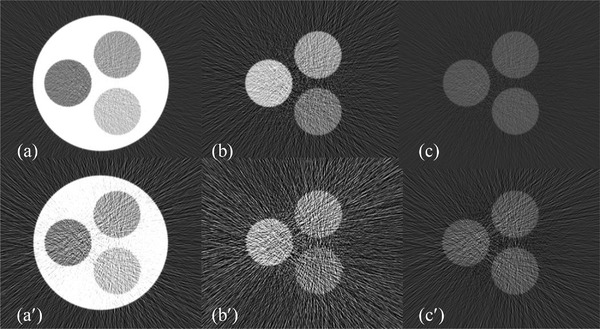
Material‐specific (tissue [left], 10 mg/ml I [middle] and bone [right]) images of the noise correlation phantom acquired via three‐material decomposition (3‐MD), whereas the detector response is ideal (a–c) or realistic (a′–c′)

The material‐specific images corresponding to basis materials soft tissue, 10 mg/ml iodine, and cortical bone acquired via 3‐MD under ideal detector spectral response are presented in Figure 5a–c. Overall, given identical X‐ray dose, the basis images acquired via 3‐MD (Figure 5) are noisier than those via 2‐MD (Figure 3), as fewer X‐ray photons fall into each spectral channel in the former than that in the latter. It is also noted that the interchannel overlap in detector's response increases the magnitude of noise in each material‐specific image significantly (Figure 5), which is within anticipation according to Equations (A1)–(A3).

**FIGURE 6:**
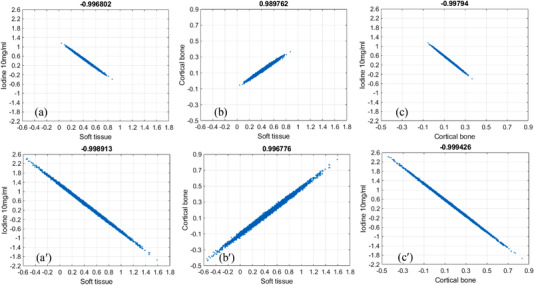
Correlation of noise between the material‐specific images acquired via three‐material decomposition (3‐MD) under both ideal (a–c) and realistic (a′–c′) detector response

The correlation of noise in the material‐specific images corresponding to basis materials soft tissue, 10 mg/ml iodine, and cortical bone under ideal detector spectral response are presented in the scatterplots in Figure 6a–c. The polarity of noise correlation over the material‐specific images in 3‐MD‐based spectral imaging alternates between ±1. The interchannel overlap induced by the spectral distortion in a realistic detector's spectral response increases the magnitude of noise correlation modestly, which is again within expectation according to Equations (A1′)–(A3′).

### 3.3 Noise and noise correlation in 4‐MD material‐specific images

**FIGURE 7:**
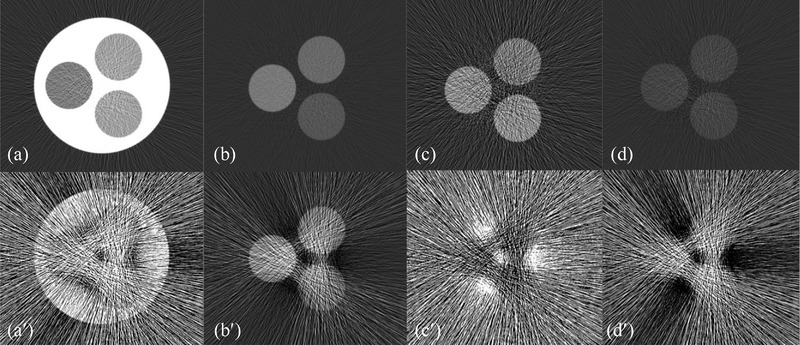
Material‐specific (left to right: soft tissue, [10 mg/ml] Gd, [10 mg/ml] I and cortical bone) images of the noise correlation phantom acquired via four‐material decomposition (4‐MD) under ideal (a–d) and realistic (a′–d′) detector response

The material‐specific images corresponding to basis materials soft tissue, 10 mg/ml gadolinium, 10 mg/ml iodine, and cortical bone via 4‐MD under ideal detector spectral response are displayed in Figure 7a–c. Overall, given the same X‐ray dose, the noise in the 4‐MD basis images is much stronger than that in the 3‐MD case, as the number of X‐ray photons falling into each spectral channel is further fewer, as demonstrated by visual comparison between Figures 5a–c and 7a–d. Similar to what has been observed in the cases of 2‐MD and 3‐MD, the interchannel overlap in detector's response in 4‐MD makes the noise in each material‐specific image much stronger (Figure 7a′–d′) over their counterparts under ideal detector spectral response (Figure 7a′–d′).

**FIGURE 8:**
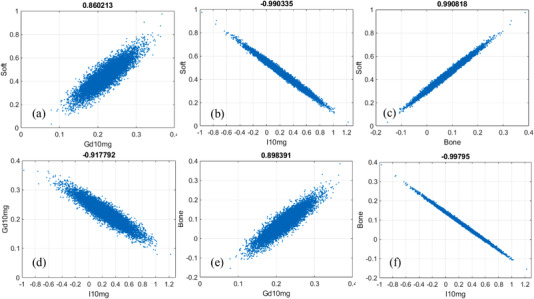
Noise correlation in four‐material decomposition (4‐MD): (a) soft tissue versus gadolinium, (b) soft tissue versus iodine, (c) soft tissue versus bone, (d) gadolinium versus iodine, (e) gadolinium versus bone, and (f) iodine versus bone

**FIGURE 9:**
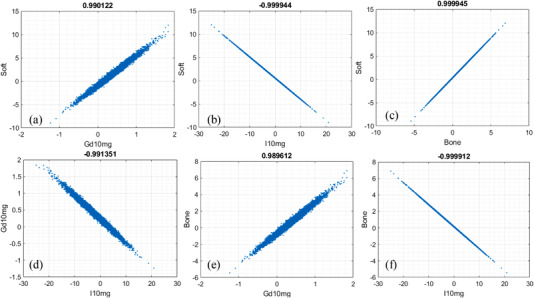
Noise correlation in four‐material decomposition (4‐MD): (a) soft tissue versus gadolinium, (b) soft tissue versus iodine, (c) soft tissue versus bone, (d) gadolinium versus iodine, (e) gadolinium versus bone, and (f) iodine versus bone

The correlation of noise between the material‐specific images in 4‐MD are presented in the scatterplots in Figure 8a–f. Similar to the cases in 3‐MD, the polarity of noise correlation between those material‐specific images in 4‐MD alternates between ±1. Obviously, the interchannel spectral overlap induced by the spectral distortion in realistic detector's spectral response in 4‐MD increases the magnitude of noise correlation considerably (compare Figures 8a–f and 9a–f), similar to what happens in the cases of 2‐MD and 3‐MD. Moreover, it is observed in Figure 7 that the noise in the material‐specific image corresponding to gadolinium (Figure 7c,c′) is the strongest in comparison to other material‐specific images. Consequently, the Person correlation coefficient decreases significantly whenever gadolinium is involved, as illustrated in Figure 8a,d,e in comparison to Figures 8b,c,f, and 9a,d,e compared to Figure 8b,c,f as well. We believe that the underlying reason is that gadolinium is much more attenuating than other materials in the phantom. In particular, the *K*‐edge of gadolinium is at 50.2 keV, which is very close to the effective energy of the polychromatic X‐ray source utilized in the simulation study.

### 3.4 Impact of noise correlation on the performance of spectral imaging

**FIGURE 10:**
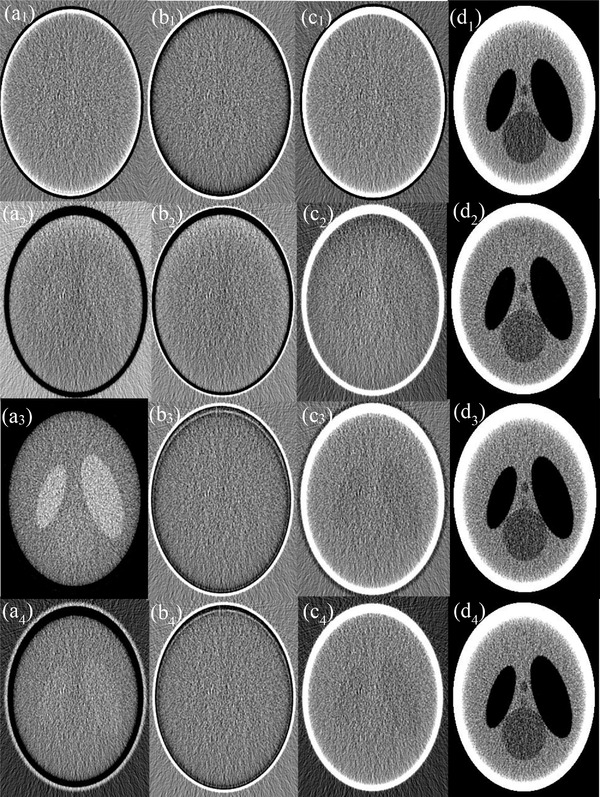
Basis images (columns 1–3) of the head phantom acquired with basis material sets BM‐1, BM‐2, BM‐3, and BM‐4 (rows 1–4) and resultant virtual monochromatic images (VMIs) at 45 keV (column 4) under ideal detector response

**FIGURE 11:**
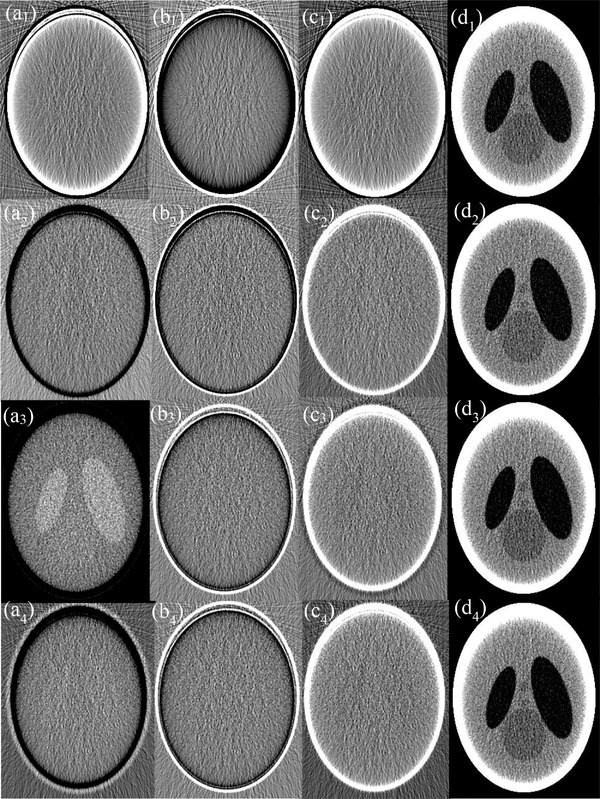
Basis images (columns 1–3) of the head phantom acquired with basis material sets BM‐1, BM‐2, BM‐3, and BM‐4 (rows 1–4) and resultant virtual monochromatic images (VMIs) at 45 keV (column 4), under realistic detector response

Shown in the first row of Figure 10 are material‐specific images of the head phantom (columns 1–3) acquired via 3‐MD with basis material set BM‐1 and the virtual monochromatic image (VMI) formed at 45 keV (column 4) under ideal detector spectral response, whereas those in rows 2–4 are their counterparts acquired with basis material sets BM‐2, BM‐3, and BM‐4. It is observed that, in contrast to the drastic variation of noise in the material‐specific images over the four basis material sets, the noise in the VMIs formed at 45 keV only varies modestly. Similar observation can be made on their counterparts acquired under realistic detector spectral response (Figure 11).

**FIGURE 12:**
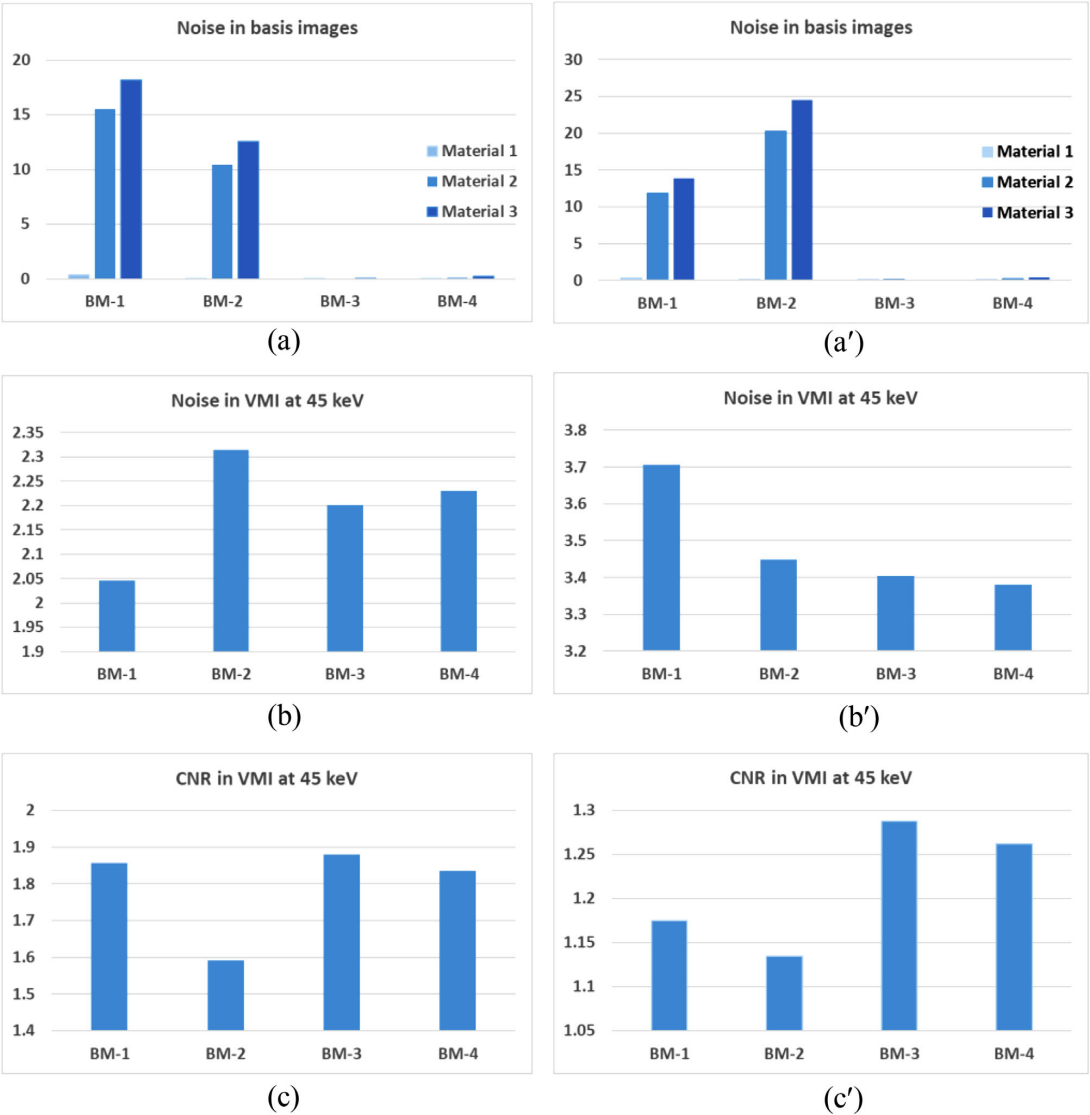
Noise in the material‐specific images of each basis material set (row 1), and the resultant noise (row 2) and contrast‐to‐noise ratio (CNR) (row 3) in each virtual monochromatic image at 45 keV under ideal (left) and realistic (right) detector spectral response

The noises gauged in each of the material‐specific images in Figure 10 under ideal detector spectral response are charted in Figure 12a, showing that the variation of noise within the basis material sets BM‐1 and BM‐2 is drastic. Similar phenomenon is observed under realistic detector spectral response (Figures 11 and 12a′), except for the markedly increased magnitude in noise. Moreover, an inspection of Figure 12a,a′ reveals that the variation of noise in the material‐specific images across the basis material sets BM‐1, BM‐2, BM‐3, and BM‐4 is drastic too. On the other hand, under both ideal and realistic detector spectral response, the noise in the VMIs at 45 keV only varies modestly across the four basis material sets (Figure 12b,b′), leading to moderate variation in the CNR (Figure 12c,c′) that is consistent to visual inspection of the images displayed in Figures 10d_1_–d_4_ and 11d_1_–d_4_.

## 4 DISCUSSION

Recognizing the momentum in R&D of photon‐counting CT and the potential of *m*‐MD‐based spectral imaging, we derived the equations governing the behavior of noise and noise correlation in the material‐specific images for spectral imaging in photon‐counting CT based on *m*‐MD. As the analytic expression for noise and noise correlation in the cases beyond 2‐MD becomes complicated, we carried out a simulation with phantoms to assess the noise correlation between the material‐specific images, and it impact on the performance of *m*‐MD‐based spectral imaging in photon‐counting CT. Specifically, by extending the study of noise correlation from 2‐MD‐based spectral imaging to the *m*‐MD‐ (3‐MD, 4‐MD and beyond) based cases in photon‐counting CT, we characterized the quantitative relation in noise correlation between the material‐specific (basis) images in *m*‐MD‐based spectral imaging, which is significantly more complicated than its counterpart in the 2‐MD‐based case. To the best of our knowledge, such an attempt in the paradigm of *m*‐MD‐based spectral imaging in photon‐counting CT has not been reported in the literature.

As reported in the literature,^13^, ^14^, ^16^ the noise correlation in the material‐specific images of 2‐MD is negative (Figure 4). However, as demonstrated in Figures 6, 8, and 9, the polarity of noise correlation in the material‐specific images of 3‐MD and 4‐MD alternates between ±1, and such an alternation makes the noise property in *m*‐MD‐based spectral imaging complicated in photon‐counting CT. Meanwhile, it may bring about the opportunity for us to design and implement sophisticated algorithms for de‐noising in *m*‐MD‐based spectral imaging in photon‐counting CT, for example, similar to what has been reported in the literature,^14^ or in particular, using neural network or deep learning–based approaches.^29^, ^30^ For example, the material‐specific images with their noise in negative correlation should be preferably utilized in de‐noising, whereas caution needs to be exercised if the images are in positive noise correlation.

In the case of 2‐MD, Equations (10)–(13) show that the magnitude of noise in each material‐specific image is jointly determined by the SNR of the data acquired in each spectral channel, the interchannel data correlation $\rho_{I_{12}}$ and $\rho_{I_{21}}$($\rho_{I_{21}}=\rho_{I_{12}}$), the effective attenuation coefficients μ_11_, μ_12_, μ_21_, and μ_22_, and the Jacobians $\Delta_{2\times 2}^{2}$ that is depending on μ_11_, μ_12_, μ_21_, and μ_22_. Similarly, in the case of 3‐MD, Equations (A1)–(A6) show that the magnitude of noise in each material‐specific image is jointly determined by the SNR of the data acquired in each spectral channel, the interchannel data correlation $\rho_{I_{k1k2}}$ $\left(k_{1}=1,2,3;k_{2}=1,2,3\right)$, the effective attenuation coefficients $\mu_{kp}$ (k=1,2,3;p=1,2,3), and the Jacobian $\Delta_{3\times 3}^{2}$ that is dependent on $\mu_{kp}$ (k=1,2,3;p=1,2,3). In the case of either 2‐MD or 3‐MD, the distortion in a realistic detector's spectral response induces interchannel data correlation, that is, $\rho_{I_{12}}=\rho_{I_{12}}>0$ or $\rho_{I_{k1k2}}$> 0 $\left(k_{1}=1,2,3;k_{2}=1,2,3\right)$. Meanwhile, the distortion in a realistic detector's spectral response also alters the effective attenuation coefficients μ_11_, μ_12_, μ_21_, and μ_22_ or $\mu_{kp}$ (k=1,2,3;p=1,2,3), and accordingly the Jacobian $\Delta_{2\times 2}^{2}$ or $\Delta_{3\times 3}^{2}$. In reality, the role played by the Jacobian in the denominator is dominant over that by the interchannel data correlation in the nominator. Consequently, the material‐specific images under realistic detector spectral response is noisier than their counterparts under ideal detector spectral response, as we have observed visually in Figures 3, 5, and 7 and quantitatively in Figure 12a–a′. Similar observation can be made on the noise in 4‐MD if we compare the results shown in Figure 7a–d and a′–d′, though the analytic equations that characterizing the noise behavior in 4‐MD is not presented due to its complexity in expression.

The polarity of noise correlation in the material‐specific images depends on their order in the set of basis materials. Specifically, the order is determined by the attenuating property of the basis materials at the low‐energy end (see Figure 12 and its related context in Ref. [6] for in‐depth analysis and discussion), which in turn determines the sign of the cofactors in getting the inverse matrixes according to Equation (5). The magnitude of noise correlation between the material‐specific images in 2‐MD and 3‐MD, regardless of its polarity, approaches unity, implying that the correlation is actually very strong. As illustrated in Equations (10)–(13) and (A1)–(A6), the interchannel data correlation ($\rho_{I_{k1k2}}$) and the effective attenuation coefficients $\mu_{kp}$ (*p*th material in *k*th energy bin) play important roles in determining the magnitude of noise correlation in 2‐MD and 3‐MD, in a manner that is similar to their influence on the magnitude of noise just explained earlier. A close inspection of Figures 4 and 6 shows that the distortion in detector's spectral response, which may be induced by charge‐sharing, the Compton scattering, and florescence‐escaping,^8^, ^9^, ^10^, ^11^, ^12^, ^13^ indeed increases the magnitude of noise correlation between the material‐specific images in comparison to their counterparts under ideal detector spectral response. Similar observation can be made on the noise correlation in the case of 4‐MD if we compare the results displayed in Figures 8a–f and 9a–f, in spite of the fact that the analytic equations that characterizing the behavior of noise correlation in 4‐MD is not explicitly given due to its complexity in expression.

The drastic variation of noise in each of the material‐specific images in 3‐MD Figure 12a,a′ is attributed to the similarity in attenuation between the basis materials adipose and PMMA and thus poor conditioning in the basis material sets BM‐1 and BM‐2.^5^, ^6^ With improvement in the conditioning of basis materials in BM‐3 and BM‐4, the variation of noise in the material‐specific images becomes much more tractable. Hence, for material‐specific imaging, attention should be paid to basis materials selection^5^, ^6^ (see Figure 12a,a′). On the other hand, despite the noise varies radically over the material‐specific images across the basis material sets BM‐1, BM‐2, BM‐3, and BM‐4 (Figure 12a,a′), the variation of noise in virtual monochromatic image is moderate (Figure 12b,b′), leading to moderate variation in the imaging performance assessed by CNR (Figure 12c,c′). It is believed that the tolerance to the conditioning of basis materials in virtual monochromatic imaging is partially attributable to the fact that there exist negative noise correlations between the material‐specific images.

Only 3‐MD and 4‐MD are considered in our study as the examples to evaluate and verify the property of noise and noise correlation in *m*‐MD. However, it should be straightforward to apply Equation (5) and its derivatives over the cases, wherein more than four basis materials are engaged in MD. Fortunately, as indicated in our recent investigation on the conditioning of basis materials and spectral channelization and their impacts on the performance of photon‐counting spectral imaging in CT,^6^, ^7^ the chance for more than four basis materials (and accordingly four spectral channels) is really limited, in light of the fact that more basis materials (and thus more spectral channels) may radically impact the imaging performance, for example, the occurrence of severe noise with increasing number of spectral channels, as we have observed in the cases presented in Figure 7a′–d′. Finally, we would like to state that the data acquired in this work are informative to the clinical medical physicists who are interested in spectral imaging in either photon‐counting or energy‐integration spectral CT. The information may also beneficiary to the clinical medical physicists who are working in other X‐ray imaging modalities, such as digital radiography and tomosynthesis, because, basically, there is no difference in carrying out the MD between them and CT.

## 5 CONCLUSIONS

By taking 3‐ and 4‐MD as the examples, we investigated the noise correlation in material‐specific (basis) images and its effect on the performance of spectral imaging in photon‐counting CT based on multi‐MD. The obtained results and conveyed information may help clinical medical physicists further understand the fundamentals of spectral imaging in either photon‐counting or energy‐integration CT based on multi‐MD, and other X‐ray imaging modalities, such as digital radiography and tomosynthesis.

## AUTHOR CONTRIBUTIONS

Xiangyang Tang: Study conception, study design, literature research, data analysis, manuscript writing, and study integrity; Yan Ren: Study design, data acquisition, data analysis, and manuscript editing; Huiqiao Xie: Data analysis and manuscript editing.

## CONFLICTS OF INTEREST

The authors have no relevant conflicts of interest to disclose.

## FUNDING INFORMATION

XT is a recipient of research grant from SinoVision Technologies, which is not relevant to the work presented herein. The authors have no other relevant financial interests to disclose.

## Data Availability

The data that support the findings of this study are available from the corresponding author upon reasonable request.

## APPENDIX A

### A.1

Starting from Equation (5) and following with algebra, we can specifically and deliberately arrive at the results presented below, though the results may be analytically expressed in a more compact form.

(A1)
$$\begin{array}{ccc} \sigma_{A_{1}A_{1}}^{2} & = & ({\left(\mu_{23}\mu_{32}-\mu_{33}\mu_{22}\right)}^{2}/\mathrm{SN}{R^{2}}_{1}\\ & & +\;{\left(\mu_{13}\mu_{32}-\mu_{33}\mu_{12}\right)}^{2}/\mathrm{SN}{R^{2}}_{2}\\ & & +\;{\left(\mu_{13}\mu_{22}-\mu_{12}\mu_{23}\right)}^{2}/\mathrm{SN}{R^{2}}_{3}\\ & & -\;2\rho_{I12}(\mu_{13}\mu_{23}{\mu^{2}}_{32}+{\mu^{2}}_{33}\mu_{12}\mu_{22}\\ & & -\;\mu_{33}\mu_{12}\mu_{23}\mu_{32}-\mu_{33}\mu_{13}\mu_{22}\mu_{32})/\mathrm{SN}R_{1}\mathrm{SN}R_{2}\\ & & -\;2\rho_{I23}({\mu^{2}}_{13}\mu_{22}\mu_{32}+\mu_{33}{\mu^{2}}_{12}\mu_{23}\\ & & -\;\mu_{12}\mu_{13}\mu_{23}\mu_{32}-\mu_{33}\mu_{12}\mu_{13}\mu_{22})/\mathrm{SN}R_{2}\mathrm{SN}R_{3}\\ & & -\;2\rho_{I13}(\mu_{12}{\mu^{2}}_{23}\mu_{32}+\mu_{33}\mu_{13}{\mu^{2}}_{22}\\ & & -\;\mu_{13}\mu_{22}\mu_{23}\mu_{32}\\ & & -\;\mu_{33}\mu_{12}\mu_{22}\mu_{23})/\mathrm{SN}R_{1}\mathrm{SN}R_{3})/{\Delta^{2}}_{3\times 3}. \end{array}$$

(A2)
$$\begin{array}{ccc} \sigma_{A_{2}A_{2}}^{2} & = & ({\left(\mu_{23}\mu_{31}-\mu_{33}\mu_{21}\right)}^{2}/\mathrm{SN}{R^{2}}_{1}\\ & & +\;{\left(\mu_{13}\mu_{31}-\mu_{33}\mu_{11}\right)}^{2}/\mathrm{SN}{R^{2}}_{2}\\ & & +\;{\left(\mu_{11}\mu_{23}-\mu_{13}\mu_{21}\right)}^{2}/\mathrm{SN}{R^{2}}_{3}\\ & & -\;2\rho_{I12}(\mu_{13}\mu_{23}{\mu^{2}}_{31}+{\mu^{2}}_{33}\mu_{11}\mu_{21}\\ & & -\;\mu_{33}\mu_{11}\mu_{23}\mu_{31}-\mu_{33}\mu_{13}\mu_{21}\mu_{31})/\mathrm{SN}R_{1}\mathrm{SN}R_{2}\\ & & -2\rho_{I23}({\mu^{2}}_{13}\mu_{21}\mu_{31}+\mu_{33}{\mu^{2}}_{11}\mu_{23}-\mu_{11}\mu_{13}\mu_{23}\mu_{31}\\ & & -\;\mu_{33}\mu_{11}\mu_{13}\mu_{21})/\mathrm{SN}R_{2}\mathrm{SN}R_{3}\\ & & -2\rho_{I13}(\mu_{11}{\mu^{2}}_{23}\mu_{31}+\mu_{33}\mu_{13}{\mu^{2}}_{21}-\mu_{13}\mu_{21}\mu_{23}\mu_{31}\\ & & -\;\mu_{33}\mu_{11}\mu_{21}\mu_{23})/\mathrm{SN}R_{1}\mathrm{SN}R_{3})/{\Delta^{2}}_{3\times 3}. \end{array}$$

(A3)
$$\begin{array}{ccc} \sigma_{A_{3}A_{3}}^{2} & = & ({\left(\mu_{11}\mu_{32}-\mu_{12}\mu_{31}\right)}^{2}/\mathrm{SN}{R^{2}}_{2}\\ & & +\;{\left(\mu_{21}\mu_{32}-\mu_{22}\mu_{31}\right)}^{2}/\mathrm{SN}{R^{2}}_{1}\\ & & +{\left(\mu_{11}\mu_{22}-\mu_{12}\mu_{21}\right)}^{2}/\mathrm{SN}{R^{2}}_{3}\\ & & -\;2\rho_{I12}(\mu_{11}\mu_{21}{\mu^{2}}_{32}+\mu_{12}\mu_{22}{\mu^{2}}_{31}\\ & & -\;\mu_{11}\mu_{22}\mu_{31}\mu_{32}-\mu_{12}\mu_{21}\mu_{31}\mu_{32})/\mathrm{SN}R_{1}\mathrm{SN}R_{2}\\ & & -\;2\rho_{I23}({\mu^{2}}_{12}\mu_{21}\mu_{31}+{\mu^{2}}_{11}\mu_{22}\mu_{32}\\ & & -\;\mu_{11}\mu_{12}\mu_{21}\mu_{32}-\mu_{11}\mu_{12}\mu_{22}\mu_{31})/\mathrm{SN}R_{2}\mathrm{SN}R_{3}\\ & & -\;2\rho_{I13}(\mu_{12}{\mu^{2}}_{21}\mu_{32}+\mu_{11}{\mu^{2}}_{22}\mu_{31}-\mu_{11}\mu_{21}\mu_{22}\mu_{32}\\ & & -\;\mu_{12}\mu_{21}\mu_{22}\mu_{31})/\mathrm{SN}R_{1}\mathrm{SN}R_{3})/{\Delta^{2}}_{3\times 3}. \end{array}$$

(A4)
$$\begin{array}{ccc} \sigma_{A_{1}A_{2}} & = & \sigma_{A_{2}A_{1}}=-(({\mu^{2}}_{23}\mu_{31}\mu_{32}+{\mu^{2}}_{33}\mu_{21}\mu_{22}\\ & & -\;\mu_{33}\mu_{21}\mu_{23}\mu_{32}-\mu_{33}\mu_{22}\mu_{23}\mu_{31})/\mathrm{SN}{R^{2}}_{1}\\ & & +\;({\mu^{2}}_{33}\mu_{11}\mu_{12}+{\mu^{2}}_{13}\mu_{31}\mu_{32}-\mu_{33}\mu_{11}\mu_{13}\mu_{32}\\ & & -\;\mu_{33}\mu_{12}\mu_{13}\mu_{31})/\mathrm{SN}{R^{2}}_{2}\\ & & +\;(\mu_{11}\mu_{12}{\mu^{2}}_{23}+{\mu^{2}}_{13}\mu_{21}\mu_{22}-\mu_{11}\mu_{13}\mu_{22}\mu_{23}\\ & & -\;\mu_{12}\mu_{13}\mu_{21}\mu_{23})/\mathrm{SN}{R^{2}}_{3}\\ & & -\;\rho_{I12}({\mu^{2}}_{33}\mu_{11}\mu_{22}+{\mu^{2}}_{33}\mu_{12}\mu_{21}\\ & & +\;2\mu_{13}\mu_{23}\mu_{31}\mu_{32}-\mu_{33}\mu_{11}\mu_{23}\mu_{32}\\ & & -\;\mu_{33}\mu_{13}\mu_{21}\mu_{32}-\mu_{33}\mu_{12}\mu_{23}\mu_{31}\\ & & -\;\mu_{33}\mu_{13}\mu_{22}\mu_{31})/\mathrm{SN}R_{1}\mathrm{SN}R_{2}\\ & & -\;\rho_{I23}({\mu^{2}}_{13}\mu_{21}\mu_{32}+2\mu_{33}\mu_{11}\mu_{12}\mu_{23}\\ & & +\;{\mu^{2}}_{13}\mu_{22}\mu_{31}-\mu_{11}\mu_{13}\mu_{23}\mu_{32}\\ & & -\;\mu_{12}\mu_{13}\mu_{23}\mu_{31}-\mu_{33}\mu_{11}\mu_{13}\mu_{22}\\ & & -\;\mu_{33}\mu_{12}\mu_{13}\mu_{21})/\mathrm{SN}R_{2}\mathrm{SN}R_{3}\\ & & -\rho_{I13}(\mu_{12}{\mu^{2}}_{23}\mu_{31}+2\mu_{33}\mu_{13}\mu_{21}\mu_{22}\\ & & +\;\mu_{11}{\mu^{2}}_{23}\mu_{32}-\mu_{13}\mu_{21}\mu_{23}\mu_{32}\\ & & -\;\mu_{13}\mu_{22}\mu_{23}\mu_{31}-\mu_{33}\mu_{11}\mu_{22}\mu_{23}\\ & & -\;\mu_{33}\mu_{12}\mu_{21}\mu_{23})/\mathrm{SN}R_{1}\mathrm{SN}R_{3})/{\Delta^{2}}_{3\times 3}. \end{array}$$

(A5)
$$\begin{array}{ccc} \sigma_{A_{1}A_{3}} & = & \sigma_{A_{3}A_{1}}=-((\mu_{21}\mu_{23}{\mu^{2}}_{32}+\mu_{33}{\mu^{2}}_{22}\mu_{31}\\ & & -\;\mu_{22}\mu_{23}\mu_{31}\mu_{32}-\mu_{33}\mu_{21}\mu_{22}\mu_{32})/\mathrm{SN}{R^{2}}_{1}\\ & & +\;(\mu_{11}\mu_{13}{\mu^{2}}_{32}+\mu_{33}{\mu^{2}}_{12}\mu_{31}-\mu_{12}\mu_{13}\mu_{31}\mu_{32}\\ & & -\;\mu_{33}\mu_{11}\mu_{12}\mu_{32})/\mathrm{SN}{R^{2}}_{2}\\ & & +\;({\mu^{2}}_{12}\mu_{21}\mu_{23}+\mu_{11}\mu_{13}{\mu^{2}}_{22}-\mu_{12}\mu_{13}\mu_{21}\mu_{22}\\ & & -\;\mu_{11}\mu_{12}\mu_{22}\mu_{23})/\mathrm{SN}{R^{2}}_{3}\\ & & -\;\rho_{I12}(\mu_{11}\mu_{23}{\mu^{2}}_{32}+2\mu_{33}\mu_{12}\mu_{22}\mu_{31}\\ & & +\;\mu_{13}\mu_{21}{\mu^{2}}_{32}-\mu_{12}\mu_{23}\mu_{31}\mu_{32}\\ & & -\mu_{13}\mu_{22}\mu_{31}\mu_{32}-\mu_{33}\mu_{11}\mu_{22}\mu_{32}\\ & & -\;\mu_{33}\mu_{12}\mu_{21}\mu_{32})/\mathrm{SN}R_{1}\mathrm{SN}R_{2}\\ & & -\rho_{I23}({\mu^{2}}_{12}\mu_{23}\mu_{31}+2\mu_{11}\mu_{13}\mu_{22}\mu_{32}\\ & & +\;\mu_{33}{\mu^{2}}_{12}\mu_{21}-\mu_{11}\mu_{12}\mu_{23}\mu_{32}\\ & & -\;\mu_{12}\mu_{13}\mu_{21}\mu_{32}-\mu_{12}\mu_{13}\mu_{22}\mu_{31}\\ & & -\;\mu_{33}\mu_{11}\mu_{12}\mu_{22})/\mathrm{SN}R_{2}\mathrm{SN}R_{3}\\ & & -\rho_{I13}(\mu_{13}{\mu^{2}}_{22}\mu_{31}+2\mu_{12}\mu_{21}\mu_{23}\mu_{32}\\ & & +\;\mu_{33}\mu_{11}{\mu^{2}}_{22}-\mu_{11}\mu_{22}\mu_{23}\mu_{32}\\ & & -\mu_{13}\mu_{21}\mu_{22}\mu_{32}-\mu_{12}\mu_{22}\mu_{23}\mu_{31}\\ & & -\;\mu_{33}\mu_{12}\mu_{21}\mu_{22})/\mathrm{SN}R_{1}\mathrm{SN}R_{3})/{\Delta^{2}}_{3\times 3}. \end{array}$$

(A6)
$$\begin{array}{ccc} \sigma_{A_{2}A_{3}} & = & \sigma_{A_{3}A_{2}}=-((\mu_{33}{\mu^{2}}_{21}\mu_{32}+\mu_{22}\mu_{23}{\mu^{2}}_{31}\\ & & -\;\mu_{21}\mu_{23}\mu_{31}\mu_{32}-\mu_{33}\mu_{21}\mu_{22}\mu_{31})/\mathrm{SN}{R^{2}}_{1}\\ & & +\;(\mu_{33}{\mu^{2}}_{11}\mu_{32}+\mu_{12}\mu_{13}{\mu^{2}}_{31}-\mu_{11}\mu_{13}\mu_{31}\mu_{32}\\ & & -\;\mu_{33}\mu_{11}\mu_{12}\mu_{31})/\mathrm{SN}{R^{2}}_{2}\\ & & +\;({\mu^{2}}_{11}\mu_{22}\mu_{23}+\mu_{12}\mu_{13}{\mu^{2}}_{21}-\mu_{11}\mu_{12}\mu_{21}\mu_{23}\\ & & -\;\mu_{11}\mu_{13}\mu_{21}\mu_{22})/\mathrm{SN}{R^{2}}_{3}\\ & & -\rho_{I12}(\mu_{12}\mu_{23}{\mu^{2}}_{31}+2\mu_{33}\mu_{11}\mu_{21}\mu_{32}\\ & & +\;\mu_{13}\mu_{22}{\mu^{2}}_{31}-\mu_{11}\mu_{23}\mu_{31}\mu_{32}\\ & & -\;\mu_{13}\mu_{21}\mu_{31}\mu_{32}-\mu_{33}\mu_{11}\mu_{22}\mu_{31}\\ & & -\;\mu_{33}\mu_{12}\mu_{21}\mu_{31})/\mathrm{SN}R_{1}\mathrm{SN}R_{2}\\ & & -\;\rho_{I23}({\mu^{2}}_{11}\mu_{23}\mu_{32}+2\mu_{12}\mu_{13}\mu_{21}\mu_{31}\\ & & +\;\mu_{33}{\mu^{2}}_{11}\mu_{22}-\mu_{11}\mu_{13}\mu_{21}\mu_{32}\\ & & -\;\mu_{11}\mu_{12}\mu_{23}\mu_{31}-\mu_{11}\mu_{13}\mu_{22}\mu_{31}\\ & & -\;\mu_{33}\mu_{11}\mu_{12}\mu_{21})/\mathrm{SN}R_{2}\mathrm{SN}R_{3}\\ & & -\rho_{I13}(\mu_{13}{\mu^{2}}_{21}\mu_{32}+2\mu_{11}\mu_{22}\mu_{23}\mu_{31}\\ & & +\;\mu_{33}\mu_{12}{\mu^{2}}_{21}-\mu_{11}\mu_{21}\mu_{23}\mu_{32}\\ & & -\;\mu_{12}\mu_{21}\mu_{23}\mu_{31}-\mu_{13}\mu_{21}\mu_{22}\mu_{31}\\ & & -\;\mu_{33}\mu_{11}\mu_{21}\mu_{22})/\mathrm{SN}R_{1}\mathrm{SN}R_{3})/{\Delta^{2}}_{3\times 3}. \end{array}$$

where

(A7)
$$\begin{array}{ccc} \Delta_{3\times 3} & = & \mu_{11}\mu_{23}\mu_{32}-\mu_{13}\mu_{21}\mu_{32}-\mu_{12}\mu_{23}\mu_{31}\\ & & +\;\mu_{13}\mu_{22}\mu_{31}-\mu_{33}\mu_{11}\mu_{22}+\mu_{33}\mu_{12}\mu_{21}. \end{array}$$

If no correlation exists in projection data, $\left(\rho_{I_{\mathrm{ij}}}=0,i=1,2,3,4;j=1,2,3,4\right)$, Equations (A1)–(A6) degenerate into

(A1′)
$$\begin{array}{ccc} \sigma_{A_{1}A_{1}}^{2} & = & ({\left(\mu_{23}\mu_{32}-\mu_{33}\mu_{22}\right)}^{2}/\mathrm{SN}{R^{2}}_{1}\\ & & +\;{\left(\mu_{13}\mu_{32}-\mu_{33}\mu_{12}\right)}^{2}/\mathrm{SN}{R^{2}}_{2}\\ & & +\;{\left(\mu_{13}\mu_{22}-\mu_{12}\mu_{23}\right)}^{2}/\mathrm{SN}{R^{2}}_{3})/{\Delta^{2}}_{3\times 3}. \end{array}$$

(A2′)
$$\begin{array}{ccc} \sigma_{A_{2}A_{2}}^{2} & = & ({\left(\mu_{23}\mu_{31}-\mu_{33}\mu_{21}\right)}^{2}/\mathrm{SN}{R^{2}}_{1}\\ & & +\;{\left(\mu_{13}\mu_{31}-\mu_{33}\mu_{11}\right)}^{2}/\mathrm{SN}{R^{2}}_{2}\\ & & +\;{\left(\mu_{11}\mu_{23}-\mu_{13}\mu_{21}\right)}^{2}/\mathrm{SN}{R^{2}}_{3})/{\Delta^{2}}_{3\times 3}. \end{array}$$

(A3′)
$$\begin{array}{ccc} \sigma_{A_{3}A_{3}}^{2} & = & ({\left(\mu_{11}\mu_{32}-\mu_{12}\mu_{31}\right)}^{2}/\mathrm{SN}{R^{2}}_{2}\\ & & +\;{\left(\mu_{21}\mu_{32}-\mu_{22}\mu_{31}\right)}^{2}/\mathrm{SN}{R^{2}}_{1}\\ & & +\;{\left(\mu_{11}\mu_{22}-\mu_{12}\mu_{21}\right)}^{2}/\mathrm{SN}{R^{2}}_{3})/{\Delta^{2}}_{3\times 3}. \end{array}$$

(A4′)
$$\begin{array}{ccc} \sigma_{A_{1}A_{2}} & = & \sigma_{A_{2}A_{1}}=-(({\mu^{2}}_{23}\mu_{31}\mu_{32}+{\mu^{2}}_{33}\mu_{21}\mu_{22}\\ & & -\;\mu_{33}\mu_{21}\mu_{23}\mu_{32}-\mu_{33}\mu_{22}\mu_{23}\mu_{31})/\mathrm{SN}{R^{2}}_{1}\\ & & +\;({\mu^{2}}_{33}\mu_{11}\mu_{12}+{\mu^{2}}_{13}\mu_{31}\mu_{32}-\mu_{33}\mu_{11}\mu_{13}\mu_{32}\\ & & -\;\mu_{33}\mu_{12}\mu_{13}\mu_{31})/\mathrm{SN}{R^{2}}_{2}\\ & & +\;(\mu_{11}\mu_{12}{\mu^{2}}_{23}+{\mu^{2}}_{13}\mu_{21}\mu_{22}-\mu_{11}\mu_{13}\mu_{22}\mu_{23}\\ & & -\;\mu_{12}\mu_{13}\mu_{21}\mu_{23})/\mathrm{SN}{R^{2}}_{3})/{\Delta^{2}}_{3\times 3}. \end{array}$$

(A5′)
$$\begin{array}{ccc} \sigma_{A_{1}A_{3}} & = & \sigma_{A_{3}A_{1}}=-((\mu_{21}\mu_{23}{\mu^{2}}_{32}+\mu_{33}{\mu^{2}}_{22}\mu_{31}\\ & & -\;\mu_{22}\mu_{23}\mu_{31}\mu_{32}-\mu_{33}\mu_{21}\mu_{22}\mu_{32})/\mathrm{SN}{R^{2}}_{1}\\ & & +\;(\mu_{11}\mu_{13}{\mu^{2}}_{32}+\mu_{33}{\mu^{2}}_{12}\mu_{31}-\mu_{12}\mu_{13}\mu_{31}\mu_{32}\\ & & -\;\mu_{33}\mu_{11}\mu_{12}\mu_{32})/\mathrm{SN}{R^{2}}_{2}\\ & & +\;({\mu^{2}}_{12}\mu_{21}\mu_{23}+\mu_{11}\mu_{13}{\mu^{2}}_{22}-\mu_{12}\mu_{13}\mu_{21}\mu_{22}\\ & & -\;\mu_{11}\mu_{12}\mu_{22}\mu_{23})/\mathrm{SN}{R^{2}}_{3})/{\Delta^{2}}_{3\times 3}. \end{array}$$

(A6′)
$$\begin{array}{ccc} \sigma_{A_{2}A_{3}} & = & \sigma_{A_{3}A_{2}}=-((\mu_{33}{\mu^{2}}_{21}\mu_{32}+\mu_{22}\mu_{23}{\mu^{2}}_{31}\\ & & -\;\mu_{21}\mu_{23}\mu_{31}\mu_{32}-\mu_{33}\mu_{21}\mu_{22}\mu_{31})/\mathrm{SN}{R^{2}}_{1}\\ & & +\;(\mu_{33}{\mu^{2}}_{11}\mu_{32}+\mu_{12}\mu_{13}{\mu^{2}}_{31}-\mu_{11}\mu_{13}\mu_{31}\mu_{32}\\ & & -\;\mu_{33}\mu_{11}\mu_{12}\mu_{31})/\mathrm{SN}{R^{2}}_{2}\\ & & +\;({\mu^{2}}_{11}\mu_{22}\mu_{23}+\mu_{12}\mu_{13}{\mu^{2}}_{21}-\mu_{11}\mu_{12}\mu_{21}\mu_{23}\\ & & -\;\mu_{11}\mu_{13}\mu_{21}\mu_{22})/\mathrm{SN}{R^{2}}_{3})/{\Delta^{2}}_{3\times 3}. \end{array}$$

## References

[1] Mettler F , Mahesh M , Chatfield M , et al. National Council on Radiation Protection and Measurements, Report No. 184 – Medical Radiation Exposure of patients in the United States. ISBN 9781944888169. Bethesda, MD; 2019. https://ncrponline.org/shop/reports/report‐no‐184‐medical‐radiation‐exposure‐of‐patients‐in‐the‐united‐states‐2019/

[2] Bae KT . Intravenous contrast medium administration and scan timing at CT: considerations and approaches. Radiology. 2010;256(1):32‐61. 20574084 10.1148/radiol.10090908

[3] Lusic H , Grinstaff MW . X‐ray computed tomography contrast agents. Chem Rev. 2013;113:1641‐1666. 23210836 10.1021/cr200358s PMC3878741

[4] Zhou Z , Ren L , Rajendran K , et al. Simultaneous dual‐contrast imaging using energy‐integrating detector multi‐energy CT: an in vivo feasibility study. Med Phys. 2022;49(3):1458‐1467. 35018658 10.1002/mp.15448 PMC8917049

[5] Ren Y , Xie H , Long W , Yang X , Tang X . Optimization of basis material selection and energy binning in three material decomposition for spectral imaging without contrast agents in photon‐counting CT. SPIE Proc. 2020;11312:113124X. 10.1117/12.2549678

[6] Tang X , Ren Y . On the conditioning of basis materials and its impact on multi‐material decomposition based spectral imaging in photon‐counting CT. Med Phys. 2021;48(3):1100‐1116. 33411350 10.1002/mp.14708

[7] Ren Y , Xie H , Long W , Yang X , Tang X . On the conditioning of spectral channelization (energy binning) and its impact on multi‐material decomposition based spectral imaging in photon‐counting CT. IEEE Trans Biomed Eng. 2021;68(9):2678‐2688. 33385309 10.1109/TBME.2020.3048661

[8] Alvarez RE . Extraction of Energy‐Dependent Information in Radiography. (Doctoral dissertation) Stanford University; 1976.

[9] Alvarez RE , Macovski A . Energy‐selective reconstructions in X‐ray computerised tomography. Phys Med Biol. 1976;21(5):733‐744. 967922 10.1088/0031-9155/21/5/002

[10] Alvarez RE . Dimensionality and noise in energy selective X‐ray imaging. Med Phys. 2013;40(11):111909. 24320442 10.1118/1.4824057 PMC3808483

[11] Kelcz F , Joseph PM , Hilal SK . Noise considerations in dual energy CT scanning. Med Phys. 1979;6(5):418‐425. 492076 10.1118/1.594520

[12] Primak AN , Ramirez Giraldo JC , Liu X , Yu L , McCollough CH . Improved dual‐energy material discrimination for dual‐source CT by means of additional spectral filtration. Med Phys. 2009;36(4):1359‐1369. 19472643 10.1118/1.3083567 PMC2719491

[13] Alvarez R , Seppi E . A comparison of noise and dose in conventional and energy selective computed tomography. IEEE Trans Nucl Sci. 1979;26(2):2853‐2856.

[14] Kalender WA , Klotz E , Kostaridou L . Algorithm for noise suppression in dual energy CT material density images. IEEE Trans Med Imaging. 1988;7(3):218‐224. 18230472 10.1109/42.7785

[15] Sones RA , Barnes GT . Noise correlation in image acquired simultaneously with a dual‐energy sandwich detector. Med Phys. 1989;16(6):858‐861. 2685530 10.1118/1.596311

[16] Roessl E , Ziegler A , Proksa R . On the influence of noise correlations in measurement data on basis image noise in dual‐energylike X‐ray imaging. Med Phys. 2007;34(3):959‐966. 17441242 10.1118/1.2514058

[17] Tang X , Ren Y . Noise correlation in multi‐material decomposition‐based spectral imaging in photon‐counting CT. Proceedings of the 16th Virtual International Meeting on Fully 3D Image Reconstruction in Radiology and Nuclear Medicine. 2021:414‐418. Fully 3D

[18] Ahrens JH , Dieter U . Computer generation of Poisson deviates, from modified normal distributions. ACM Trans Math Softw. 1982;8(2):163‐179

[19] Keh‐Shih C , Huang HK . Comparison of four dual energy image decomposition methods. Phys Med Biol. 1988;33(4):455.

[20] Goodsitt MM , Christodoulou EG , Larson SC . Accuracies of the synthesized monochromatic CT numbers and effective atomic numbers obtained with a rapid kVp switching dual energy CT scanner. Med Phys. 2011;38(4):2222‐2232. 21626956 10.1118/1.3567509

[21] De Man B , Basu S , Naveen C , et al. CatSim: a new computer assisted tomography simulation environment. SPIE Proc. 2007;6510:65102G. 10.1117/12.710713

[22] Chen H , Xu C , Persson M , Danielsson M . Optimization of beam quality for photon‐counting spectral computed tomography in head imaging: simulation study. SPIE J Med Imaging. 2015;2(4):043504. 10.1117/1.JMI.2.4.043504 PMC4718445 26835495

[23] Buzug TM . Computed Tomography: *F*rom Photon Statistics to Modern Cone‐Beam CT. 1st ed. Springer‐Verlag Berlin Heidelberg; 2008. doi: 10.1007/978-3-540-39408-2

[24] Cowan G . Statistical Data Analysis. Oxford University Press; 1998.

[25] Woodard HQ , White DR . The composition of body tissues. Br J Radiol. 1986;59(12):1209‐1219. 3801800 10.1259/0007-1285-59-708-1209

[26] White DR , Griffith RV , Wilson IJ . ICRU Report 46: photon, electron, proton and neutron interaction data for body tissues. J Int Comm Radiat Units Meas. 1992;24(1). https://journals.sagepub.com/toc/cru/os-24/1

[27] Cullen DE , Hubbell JH , Kissel L . EPDL97: The Evaluated Photon Data Library. Lawrence Livermore National Laboratory Report UCRL‐LR‐50400 vol 6, rev 5. Sep. 19, 1997. https://www.osti.gov/servlets/purl/295438

[28] Zhang S , Han D , Politte DG , Williamson JF , O'Sullivan JA . Impact of joint statistical dual‐energy CT reconstruction of proton stopping power images: comparison to image‐ and sinogram‐domain material decomposition approaches. Med Phys. 2018;45(5):2129‐2142. 29570809 10.1002/mp.12875 PMC6519929

[29] Gao Z , Wang X , Sun S , et al. Learning physical properties in complex visual scenes: an intelligent machine for perceiving blood flow dynamics from static CT angiography imaging. Neural Netw. 2020;123(3):82‐93. 31835156 10.1016/j.neunet.2019.11.017

[30] Charbonnier J‐P , van Rikxoort EM , Setio AAA , Schaefer‐Prokop CM , Ginneken Bvan , Ciompi F . Improving airway segmentation in computed tomography using leak detection with convolutional networks. Med Image Anal. 2017;36(2):52‐60. 27842236 10.1016/j.media.2016.11.001

